# Plasmonic nanoprinting: thinner, brighter & swifter

**DOI:** 10.1038/s41377-022-01069-z

**Published:** 2023-01-18

**Authors:** Lei Jin, Cheng-Wei Qiu

**Affiliations:** 1 Key Laboratory of RF Circuits & System of Ministry of Education, School of Electronics and Information, Hangzhou Dianzi University, Xiasha High Education Park, Hangzhou, 310018 China; 2 Department of Electrical and Computer Engineering, National University of Singapore, 4 Engineering Drive 3, Singapore, 117583 Singapore

**Keywords:** Optical materials and structures, Displays

*Nature Nanotechnology* (2022) 10.1038/s41565-022-01256-4

The plasmonic nanoprinting displays fade-resistant colours with ultrahigh resolution and opens a new avenue toward the digital colour display. An ideal product of nanoprinting requires low-cost production, high-saturation performance and dynamic tunability, whereas it is still challenging to achieve all these features simultaneously. A research team led by Prof. Yan-qing Lu and Prof. Ting Xu from Nanjing University utilised plasmonic metasurfaces formed by shallow nanoapertures to realise high-performance full-colour nanopainting. The nanoaperture is thin enough to be fabricated by reusable template-stripping technique, which guarantees decent quality of metasurfaces. By judiciously controlling the geometries and orientation of nanoapertures, such metasurfaces showed high-colour saturation, wide gamut palette and ultra-smooth brightness transition, and demonstrated photorealistic and stereoscopic full-colour painting. Moreover, such pixelated metasurface can encrypt kaleidoscopic information, the state of which could be decrypted via polarisations of incident and reflected light. Thanks to these advancements, coloration technologies could now be fabricated at a faster speed and lower cost, yet well maintaining the high-quality chiaroscuro presentation, versatile polarisation tunability, and impressive brightness. It may power up a wide range of photonic and optoelectronic applications.

